# *Mycobacterium abscessus* pulmonary infection and associated respiratory function in cystic fibrosis-like βENaC mice

**DOI:** 10.3389/ftubr.2024.1473341

**Published:** 2024-10-30

**Authors:** Camron M. Pearce, Timothy D. Shaw, Brendan Podell, Mary Jackson, Marcela Henao-Tamayo, Andres Obregon-Henao, Ha Lam, Ilham M. Alshiraihi, Wanda O’Neal, Alessandra Livraghi-Butrico, Anthony J. Hickey, Bernd Meibohm, Mercedes Gonzalez-Juarrero

**Affiliations:** 1 Cell and Molecular Biology, Colorado State University, Fort Collins, CO, United States; 2 Microbiology, Immunology and Pathology, Mycobacteria Research Laboratories, NTM Center, Colorado State University, Fort Collins, CO, United States; 3 Wellcome-Wolfson Institute for Experimental Medicine, Queen’s University Belfast, Belfast, United Kingdom; 4 Marsico Lung Institute, University of North Carolina at Chapel Hill, Chapel Hill, NC, United States; 5 RTI International, Research Triangle Park, Durham, NC, United States; 6 Department of Pharmaceutical Sciences, College of Pharmacy, University of Tennessee Health Science Center, Memphis, TN, United States

**Keywords:** *Mycobacterium abscessus*, cystic fibrosis, mouse models, βENaC-Tg, respiratory function

## Abstract

**Introduction:**

Chronic pulmonary infection with *Mycobacterium abscessus* (*M. abscessus*) is a significant cause of morbidity and mortality in people with cystic fibrosis (CF). Developing an animal model of *M. abscessus* pulmonary infection, especially under CF conditions, is essential to understanding clinical pulmonary *M. abscessus* infection. βENaC transgenic mice are known to develop spontaneous CF-like disease characterized by airway mucus obstruction and inflammation. The aim of this study was to evaluate the suitability of βENaC mice as a preclinical model and characterize their respiratory function during *M. abscessus* lung infection.

**Methods:**

Mice received an intrapulmonary aerosol of *M. abscessus* using a high-pressure syringe device (Penn-Century) for subsequent characterization of disease progression and respiratory function. Whole body unrestrained plethysmography (WBP) data was collected to monitor lung function and endpoints determined organ bacterial burden and associated pathology.

**Results:**

Endpoint CFU data in the lung and spleen showed that there was no significant difference in bacterial clearance between βENaC and WT mice. WBP data showed an impairment in overall respiratory function during and after *M. abscessus* infection in both strains of mice. Interestingly, even in wildtype control mice, lung dysfunction persisted after bacterial clearance.

**Discussion:**

Even with CF-like features, the βENaC transgenic mice cleared *M. abscessus* at a similar rate than WT mice, however, the associated respiratory monitoring revealed that there are long-term implications of *M. abscessus* lung exposure. The clear decline in respiratory function, even after *M. abscessus* clearance, suggests that WBP coupled animal modeling provides important insight that is relevant to disease burden and treatment efficacy. The *M. abscessus* clearance in the βENaC mice may help improve the fields understanding of CF-modulated immune deficiencies in *M. abscessus* pulmonary infection.

## 1 Introduction

*Mycobacterium abscessus* (*M. abscessus*) is a non-tuberculous mycobacterium (NTM) with the capacity to cause severe chronic pulmonary infections. While typically considered opportunistic, *M. abscessus* has been isolated from patients without obvious risk factors, but its prevalence is highest among individuals with pre-existing lung conditions, like cystic fibrosis (CF) (1, 2). CF is caused by a variety of different mutations in the cystic fibrosis transmembrane conductance regulator (CFTR) and is characterized by the predictable development of mucus obstruction, unconventional inflammation, and chronic bacterial infection of the lungs (3, 4). Although other bacterial species are more commonly isolated from CF patient infections, *M. abscessus* infection poses its own set of challenges, namely immune-mediated inflammation, antibiotic resistance, intracellular persistence, and biofilm formation that all contribute to a particularly severe chronic and destructive lung disease (5, 6). In the context of CF, *M. abscessus* infections are associated with rapid declines in respiratory function (7, 8), resulting in increased mortality rates and complications for lung transplantation (9).

Unfortunately, the lack of a representative animal model that is permissive to *M. abscessus* chronic infection has impeded progress in preclinical research (10). Immunocompromised mouse models have been established for *M. abscessus* infection, but are not representative or as well-characterized as other models of pulmonary mycobacterial infections, namely *M. tuberculosis* (11–15). Current *M. abscessus* infection models lack the ability to study factors such as pathogenesis, manifestation, and therapeutic efficiency in an immune competent host, which is further complicated in the context of CF, where CFTR mutant mice are hindered by their high susceptibility to life-threatening intestinal obstruction, short life span and failure to develop spontaneous lung disease (16, 17). There are no fully validated chronic animal models of Mab models of chronic infection [(18), under review]. Recent studies have validated the potential of delivering immobilizing agents such as *M. abscessus* loaded agar beads or dexamethasone-induced immunosuppression to promote pulmonary persistence and pathological response in an immunocompetent mouse (14, 19), but the development of a CF-like animal model is essential for parsing out the key mechanisms of CF host susceptibility to *M. abscessus* (20).

In βENaC transgenic (βENaC) mice, hyperactivity of the lung epithelium sodium channel (ENaC), through airway targeted overexpression of its beta subunit (βENaC, encoded by the *Scnn1b* gene), results in spontaneous disease characterized by mucus hypersecretion and chronic airway inflammation (21). Previous work shows that this accelerated Na^+^ transport alone is enough to produce CF-like lung disease in mice (22). βENaC mice exhibit spontaneous postnatal mortality which varies depending on the genetic background and is limited to 20% in congenic C57Bl6/N βENaC mice (23). The βENaC mouse has since provided a model to study mucociliary dysfunction, lung neutrophilia, neutrophil extracellular trap (NET) formation, and infection of other opportunistic pathogens such as *Pseudomonas aeruginosa* (24–27). However, no previous studies have investigated the respiratory function of βENaC mice in the context of mycobacterial infection. Our study aimed to address this knowledge gap by utilizing Whole Body Plethysmography (WBP) to assess respiratory function in wildtype (WT) and βENaC mice during *M. abscessus* infection. WBP chambers provide a non-invasive method for measuring various clinically-relevant surrogate markers of respiratory function relating to respiratory effort, timing of breath segments, and bronchoconstriction (28). By combining the unique βENaC mouse model with WBP chamber analysis, our study investigates the direct impacts of *M. abscessus* infection on respiratory function in mice, while offering valuable insights into the pre-clinical presentation of infection in a CF-like mouse model.

## 2 Materials and methods

### 2.1 Animals

βENaC mice were obtained from the Animal Models Core at the Marsico Institute or from Taconic (C57BL6/N; background mouse strain for βENaC) and bred at Colorado State University. βENaC mice were maintained as hemizygous and each offspring genotyped as previously described (22). βENaC-Tg animals were identified, and ear punched for study classification. Male and female mice (*n* = 36), averaging 8 weeks of age, were weighed, and divided into randomized groups for the study. All animal protocols and procedures were approved by the Institutional Animal Care and Use Committees (IACUC) of Colorado State University.

### 2.2 Bacteria

*Mycobacterium abscessus subsp. abscessus* isolate #103, a clinically relevant rough morphology strain obtained from a cystic fibrosis patient (provided by Mary Jackson, Colorado State University), was chosen due to its relevance in a cystic fibrosis model and previous *in vitro* characterization, along with demonstrated pulmonary infection studies in other preclinical mouse models (12). Stock cultures were grown at 37°C in Middlebrook 7H9 liquid medium (HiMedia, M198-500G) supplemented with 10% OADC, 0.5% glycerol, and 0.5% tween 80. Cultures were shaken for 24-30 hours to reach exponential growth phase and removed at OD_600_ 0.6–0.8 for experimental use, as previously described (12). Culture was passed through a 26 ½G needle 15-20 times prior to dilution to minimize clumping.

### 2.3 Mouse infection

Log phase cultures were diluted into sterile 0.9% endotoxin free saline at a concentration of 2x10^7^CFU/mL. Fifty microliters of this bacterial suspension were delivered intratracheally as an intrapulmonary spray instillation to each animal using a high-pressure syringe device (PennCentury), for a targeted dose of 1 × 10^6^ CFU/lung (29). To confirm the actual bacterial deposition in the lungs, mice (*n* = 4) were sacrificed 24 h after instillation and the whole lung and spleen prepared for viable bacterial quantification which was determined by homogenizing the lung lobe and spleen using the Precellys Tissue Homogenizer (Precellys Lysing Kit, 220325-830). Thereafter, serial 5-fold dilutions of each homogenate were plated onto Middlebrook 7H11 agar (Millipore, M0428-500G) containing carbenicillin (Sigma-Aldrich, C1389-1G) and cycloheximide (GoldBio, C-930-10) and subsequently cultured for 72–96 h at 37°C until colony forming units (CFU) were visible and could be enumerated. Following infection, the mice were monitored daily for indications of weight loss or abnormal behavior requiring pre-endpoint euthanasia. The remaining groups of mice (n=4) were euthanized at each defined timepoint, and lung and spleen were enumerated for CFU and permeabilized in 4% PFA for staining and histological analysis.

### 2.4 Whole body plethysmography

Whole Body Plethysmography (WBP) allows for analysis of respiratory function in unrestrained and conscious mice. The respiratory parameters that are obtained from WBP reflect the physiological state of the lung function under different study conditions (30). WBP was performed bi-weekly for 63 days on animals (*n* = 8) from both infected and uninfected C57BL6/N (WT background) or βENaC mice. Mice were equilibrated within individual WBP chambers of a Buxco FinePointe Series Whole Body Plethysmograph (DSI Buxco respiratory solutions, Data Sciences International) for 10 min before 20 min of data acquisition. Baseline data was collected at day-3 before infection and collected for 60 days post-infection. Surrogate measures of respiratory effort and timing were taken, including respiratory frequency (f), tidal volume per breath (TVb), minute volume per breath (MVb), inspiration time (Ti), expiration time (Te), end inspiration pause (EIP) and end expiration pause (EEP). Evidence of bronchoconstriction was inferred from the enhanced pause (Penh), a composite measure previously reported in detecting murine airway resistance (31–33). A 3-point moving average for each parameter was calculated from the mean of the measured point and the preceding two points. Area under the curve (AUC) was calculated for each parameter over the study period for comparative analysis between groups. Data reports were generated in the accompanying FinePointe software and exported to GraphPad Prism v9.5.1 or R v4.3.1 for analysis.

### 2.5 Microscopy

Brightfield microscopy was done using the Vectra Polaris^®^ whole slide scanner at binned 40x magnification. Post image processing was done using Fiji ImageJ.

### 2.6 Histopathology

The right lung lobes were collected and fixed in 4% PFA (Electron Microscopy Sciences, 15714-S) in 1X PBS for subsequent paraffin embedding and sectioning at 5μm thickness. Slides were then stained with hematoxylin and eosin (H&E), Ziehl-Neelsen acid fast, and periodic acid-Schiff techniques.

### 2.7 Data handling and study limitations

The longitudinal data collection was conducted bi-weekly at a 2-s interval for 20 min per session, as well as the optimization of WBP methods (i.e., acclimation period and noise cancellation), play a crucial role in the precision and robustness of these findings. However, the absence of certain advanced analytical techniques, like signal processing, may limit the depth of understanding of respiratory dynamics and responses and its potential correlation with bacterial burden and histological analysis. The study’s focus on summary statistics and periodic sampling aligns more with a pharmacokinetic approach, which was specifically utilized to highlight WBP as a proof-of-concept. It should be emphasized that while WBP measurements encompass the entire respiratory system, including the nasal passage, it differs from pulmonary measurements obtained by other approaches of pulmonary function studies which are often terminal for each time point and do not allow for longitudinal studies. Because our main goal was to perform longitudinal studies of respiratory function in both mouse models during and after Mab infection, we chose to optimize the use of WBP. The bacterial burden reached the limits of AFB positive detection below 10^4^ CFU/mL and required too many consecutive lung sections to locate bacilli.

### 2.8 Statistics

Mouse WBP measurements were collected using the accompanying FinePointe software. The data collection spanned a 63-day period and was subsequently exported to either R v4.3.1 or GraphPad Prism v9.5.1 for further analysis. Rolling average plots, colored tables, and Euclidean clustered heatmaps were calculated and visualized using R. Analysis of the six respiratory parameters and the AUC was calculated with standard error. The AUC values were then plotted, and statistical analysis was performed using Tukey’s multiple comparisons test as part of a one-way ANOVA. In mouse *M. abscessus* modeling experiments, the bacterial burden was expressed as Log_10_CFU. Data analysis was conducted using GraphPad Prism, and statistical significance was assessed using Dunnett’s multiple comparisons test as part of a one-way ANOVA. All error bars indicate standard errors of means (SEM, *n* = 4) and significance was determined by *p* ≥ 0.05.

## 3 Results

### 3.1 *M. abscessus* clearance in βENaC and WT mice

**FIGURE 1:**
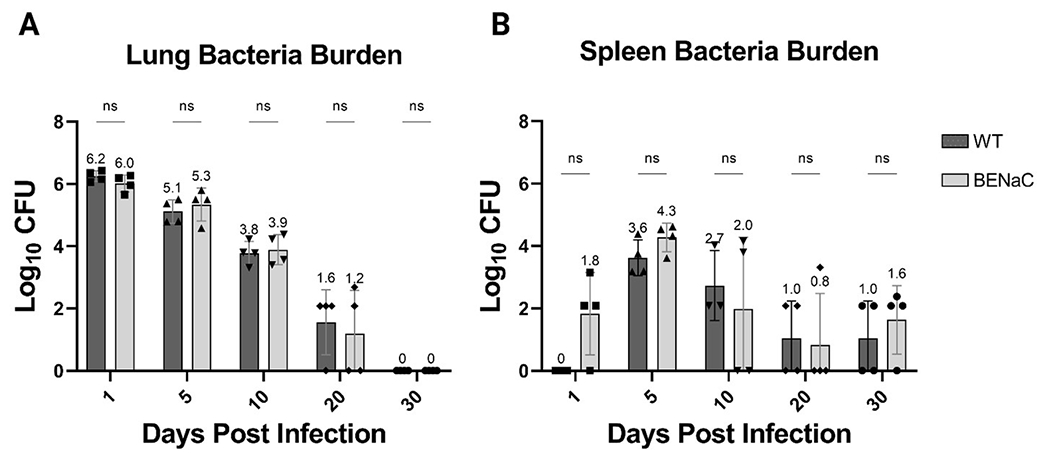
Comparison of bacterial load in the lungs and spleen of WT (dark gray) and βENaC (light gray) mice. Whole and lung adjusted **(A)**, as well as whole spleen **(B)**, Log_10_CFU values were plotted over 30 days. The y-axis represents the Log_10_CFU, while the x-axis denotes the number of days elapsed following *Mab* infection. Each column represents the mean value with associated SEM indicated by the size of the error bar. Dunnett’s multiple comparisons test: NS, Not Significant.

After intrapulmonary aerosol delivery of *M. abscessus* #103, the pulmonary and splenic bacteria load of βENaC and control WT mice were assessed at multiple timepoints (Figure 1). Immunocompetent WT mice demonstrated a 1 log-fold reduction in pulmonary bacteria load within the first 5 days, and this rate of clearance was maintained until the bacterium was undetectable on day 30 (Figure 1A). A similar but delayed trend was observed in the spleen, with *M. abscessus* appearing by day 5 and decreasing toward limits of detection (4 bacilli in the whole lung) by day 30. On day 5, 0.2% of the total pulmonary bacteria load had disseminated to the spleen, which then decreased by 10-fold every 5 days thereafter (Figure 1B). βENaC mice on day 0 of infection had similar bacterial burden in the lungs to that of the WT mice, and surprisingly, demonstrated efficient pulmonary clearance. Like the WT mice, CFU decreased 10 to 100-fold every 5 days until reaching the limit of detection of the assay by day 30 post-infection. CFU endpoints by day 30 were based on preliminary data in WT mice which indicated clearance of *M. abscessus* in both the liver and spleen after day 30 (Supplementary Figure S1). Throughout the study, mouse weights were monitored as a health indicator, and both strains of mice maintained or increased in body weight during this period (data not shown). There was no significant difference in pulmonary burden or weight change between WT and βENaC mice at any timepoint.

The spleen was evaluated for the presence of early dissemination, and 3 of 4 βENaC mice had *M. abscessus* CFUs in the spleen just 24 hours after pulmonary infection. In comparison, WT mice had no detectable splenic bacteria at 24 hours post-infection. This splenic dissemination in βENaC mice was unusually rapid and has not been observed in other mouse models, including GM-CSF KO and SCID mice (unpublished data). By day 5 post-infection, splenic dissemination had occurred in the WT mice, but the bacterial load in βENaC remained higher (median 3.6log_10_ versus 4.3log_10_), though this did not reach statistical significance. Subsequently, the reduction in splenic CFUs in both groups were similar, plateauing between days 20 and 30 close to the limits of detection.

### 3.2 Pulmonary *M. abscessus* infection modulates the respiratory waveform over time in WT and βENaC mice

**FIGURE 2:**
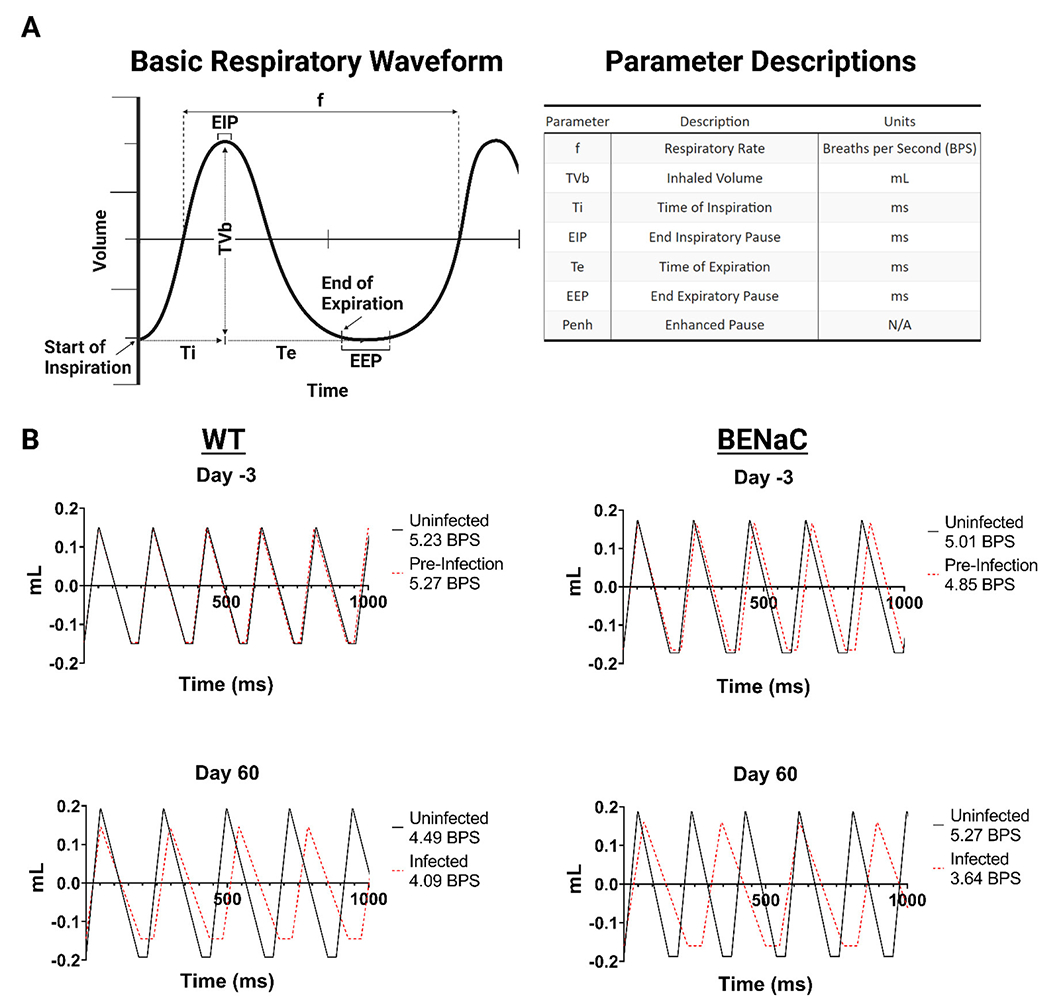
Mouse WBP respiratory waveforms before and 60 days after *Mab* challenge. **(A)** Illustration of a basic respiratory waveform to indicate WBP parameter derivation and the associated units. **(B)** Comparison of WT and βENaC respiratory waveforms with uninfected and infected animal groups overlayed in a 1,000 millisecond duration. Figure legends include the total breaths per second (BPS).

In the plethysmography studies, we first established the baseline changes in WBP that accompanied physiological aging in naïve WT and βENaC mice over the 63-day study period, and report eight key respiratory parameters including frequency (f, breaths per minute), tidal volume (TVb, volume of air per breath), minute volume (MVb, total air exchange per minute), timing parameters such as time of inspiration (Ti) and expiration (Te), end inspiratory pause (EIP, pause time after inspiration), end expiratory pause (EEP, pause time after expiration), and finally Penh (airway resistance/bronchoconstriction) (Figure 2). The relationship between each variable is important to keep in mind while reflecting on WBP respiratory data. F and TVb together determine the minute volume (MVb, Not present in Figure 2A), and a change in either parameter can influence the other and affect overall ventilation values. Ti and Te, together, define the duration of each respiratory cycle, encompassing both the EIP and EEP. Penh is a non-dimensional parameter that reflects a characteristic change in the respiratory waveform, and is traditionally used as an indicator of bronchoconstriction (32). The Penh value is influenced by changes in Te, as well as PIF and PEF, and has direct implications on the breath frequency, and by association, airway resistance.

An average respiratory waveform can be constructed using six of the 8 parameters taken from WBP data (Figure 2A). We constructed average respiratory waveforms over one second from the beginning and end timepoints of the study for each group for analysis. We sought to determine whether pulmonary Mab infection causes a global change in breathing patterns, or selectively affects breath segments. Previous murine WBP studies indicate that the transition pause times between expiration (EEP) and inspiration (EIP), are controlled by airway resistance (32). We compared the effect of Mab infection on the waveforms of WT and βENaC mice after 60 days (Figure 2B). Infected WT mice exhibited reduced tidal volume and reduced frequency compared to uninfected controls, characterized by increased expiratory time and end inspiration pause (EEP). This slower, shallower breathing pattern may be explained by physiological deconditioning in mice over time caused by chronic infection. Similar changes, but more pronounced, were observed in infected βENaC mice, who also had significantly increased Ti and Te (mean 35% for both, *p* < 0.05 and 0.01 respectively). Therefore, we found that the introduction of pulmonary *M. abscessus* modulates respiratory waveform over time, independent of the mouse genetic background, characterized by increased wavelength, decreased amplitude, and elongated breath transition times.

### 3.3 βENaC mice have impaired respiratory function compared to WT

**FIGURE 3:**
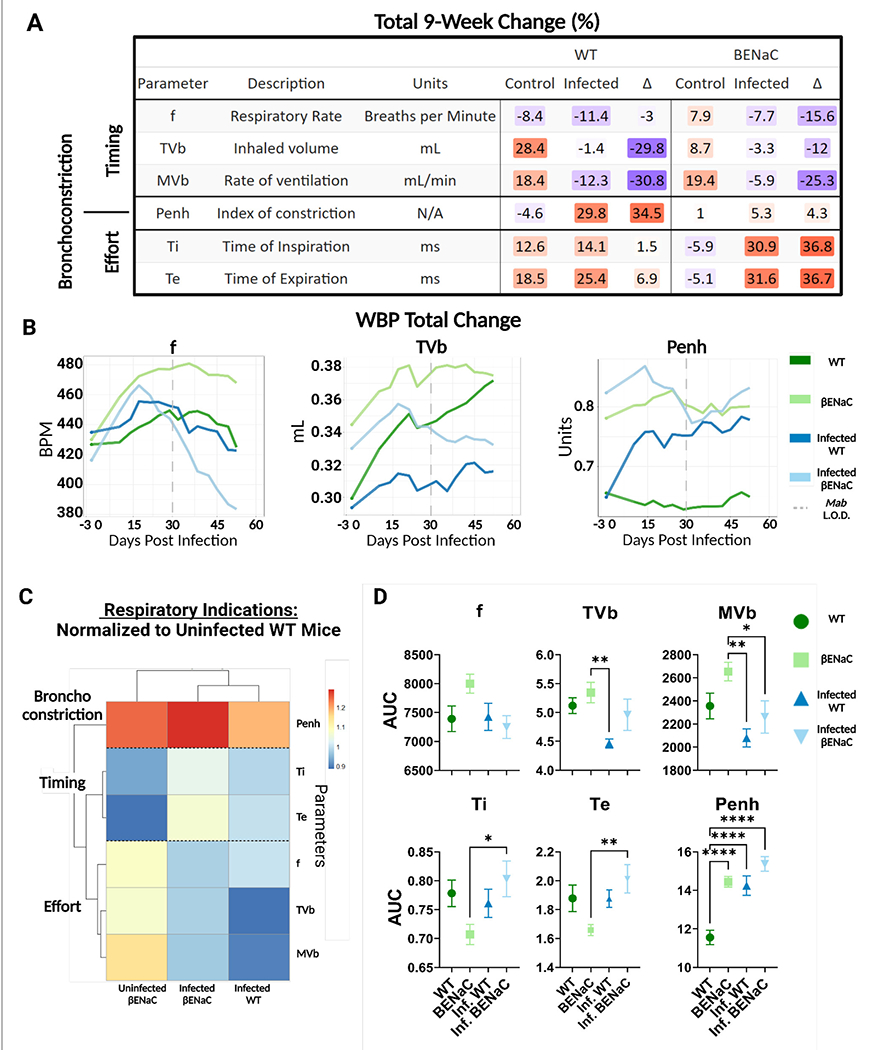
WBP respiratory measurements for WT and βENaC mice. **(A)** WBP parameters were clustered by Timing, Bronchoconstriction, and Effort. The total percent change in each parameter was calculated for both uninfected and infected conditions. The difference (Δ) between control and infected animals was calculated and change in values highlighted on a color gradient from low to high (blue to orange). **(B)** Three parameters, Penh, f, and TVb, were plotted using the rolling average over the 63-day period. The dotted line indicates when animals reached the bacterial limits of detection (LOD). **(C)** AUC values were calculated over the 63-day monitoring period and normalized to the uninfected WT mice and heatmap clustered using a Euclidean distance algorithm **(D)**. AUC values (Parameter Unit*Day) were plotted by group with individual data points indicating mean AUC value (*n* = 4), error bars showing SEM, and any changes in significance denoted by asterisks. *p < 0.05, **p < 0.01, ****p < 0.001.

Pre-infection, the average respiratory frequency was comparable between WT and βENaC mice, 5.23 vs. 5.01 breaths/second (Figure 2B), respectively, although βENaC mice had a wider SEM range between animals (0.063–0.084) compared to WT mice, which had a narrower SEM range (0.066–0.76). At baseline, βENaC mice had a higher Penh, 0.78 vs. 0.65 (βENaC intergroup SEM range 0.012–0.027 vs. WT 0.011-0.031) and tidal volume, 0.345 ml vs. WT 0.299 ml (SEM range 0.002–0.003 for both, see supplementary WBP data). This suggests βENaC mice have a higher baseline level of bronchoconstriction and increased respiratory effort compared to WT mice, indicating impaired, but not decompensated, respiratory function. Furthermore, the total change for each parameter was then calculated as a percentage for each group (Figure 3A). In naïve WT mice, frequency decreased (−7%); tidal volume increased progressively by +28.4% and inspiration/expiration times became elongated (12.6% and 18.5% respectively). These changes likely reflect physiological growth and maturation of the WT mouse. Notably, these changes were not associated with a rise in Penh, in keeping with unimpaired respiratory function.

In contrast, naïve βENaC mice had a sustained rise in frequency (+7.9%) and decreased inspiration/expiration times (−5.9% and −5.1%) over 60 days. The starting tidal volume for βENaC was ~15% higher than WT and increased by +8.7% over the study periods. Penh in control βENaC mice was elevated at baseline (approximately +20% compared to WT) but did not significantly change over time (Figure 3B). These findings suggest that impaired lung function increases modestly over time in βENaC mice but do not become decompensated.

### 3.4 Pulmonary *M. abscessus* impairs respiratory function in WT and βENaC mice even after bacterial clearance

Next, we investigated changes in respiratory function within each group caused by *M. abscessus* infection. To investigate any long-term effects associated with infection, mouse respiratory measurements were continued for 30 days after the lungs from either mouse strain no longer had culturable bacteria. In WT mice, the infection did not affect the respiratory frequency compared to uninfected controls but did dramatically impair the physiological rise in tidal volume (−1.4% compared to +28.4%) (Figure 3B). Infected WT mice also had significantly increased Penh compared to uninfected controls (29.8% vs. −4.6%), consistent with developing airway resistance. In βENaC mice frequency and tidal volume rose for the first 15 days postinfection, similarly to uninfected βENaC mice, but fell gradually over the remainder of the study period. This may be explained by an initial physiological response to lung infection, followed by physical deconditioning in chronic lung infection and progressive decompensation of respiratory function. Penh remained elevated in naïve βENaC mice (even above maximum levels detected in infected WT at any point) and did not rise further in response to infection. This may be because any bronchoconstriction generated by Mab infection in the βENaC mouse was not detectable in the background of severe constitutive airway restriction.

We then compared fundamental differences in respiratory function between the two-animal strains to further understand the effects of bacteria challenge. AUC analysis was performed for each parameter, which were clustered by similarity into three primary branches relating to (i) respiratory timing (F, Ti, Te), (ii) respiratory effort (F, TVb, MV) and bronchoconstriction (Penh) (Figure 3C). AUC values for “Infected WT”, “Uninfected βENaC” and “Infected βENaC” were normalized to the “Uninfected WT” control mice and compared for significance (Figures 3C, D). Normalized values ranged from 0.9 to 1.3, indicating that all the AUC values in the dataset were within 90%–130% of the Uninfected WT. Basal measures of respiratory function in naïve βENaC were intrinsically different to WT, characterized by lower Ti and Te, and elevated F, TVb and Penh. These values are consistent with observations of rapid and labored breathing in βENaC mice, regardless of *M. abscessus* pulmonary exposure (data not shown). AUC values from each parameter separated into unique clusters by presence or absence of infection, independent of the strains of mice; these included measures of respiratory effort which were generally impaired by infection in both WT and βENaC mice (Figure 3C). Other parameters were affected by infection to a greater degree in one strain of mice compared to the other. For instance, *M. abscessus* infection significantly increased the time of inspiration and expiration in the βENaC mice (*p* < 0.01) but not WT, whereas Penh levels were elevated significantly during infection for WT mice (*p* < 0.0001) but not βENaC. In summary, the *M. abscessus* aerosol challenge resulted in impaired respiratory function in both strains of mice, characterized by commonly shared characteristics (reduced tidal volume) and distinctive responses (increased airway resistance in WT and breath elongation in βENaC), which persisted even after clearance of pulmonary Mab.

### 3.5 βENaC mice present severe airway pathology, limiting the identification of pathogen associated features

**FIGURE 4:**
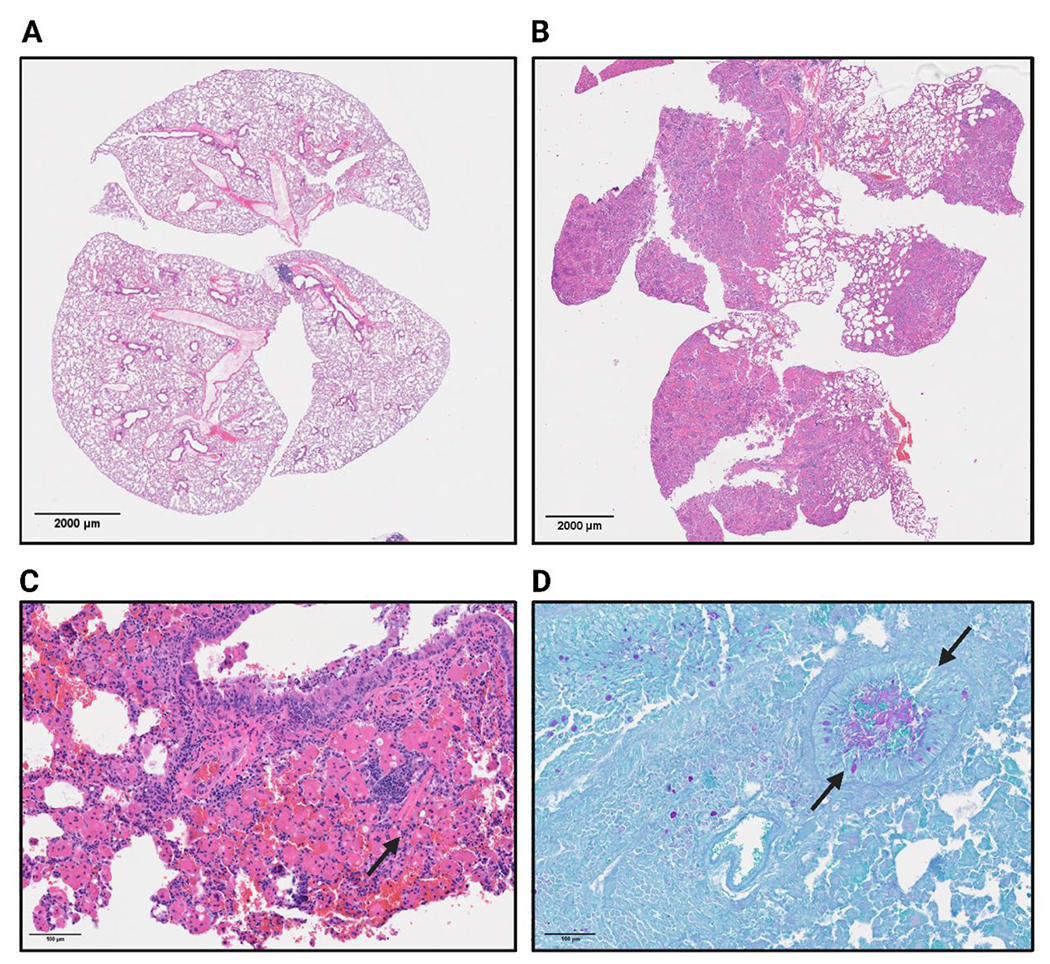
Naïve WT **(A)** and βENaC **(B)** whole lungs are represented by H&E staining to indicate areas of inflammation (lymphocytes in purple) and the general shape and structure. **(C)** Represents a 40x H&E image of a βENaC mouse with severe pathological features, of which includes the occasional eosin crystal indicated by the arrow. **(D)** Arrows show a mucus filled bronchiole in a βENaC mouse as indicated by the periodic acid oxidation of mucus buildup from the PAS staining.

Next, we examined the lungs of βENaC mice for histological correlates of infection. Postmortem gross pathology of uninfected βENaC mice presented with hyperinflated lungs with exuding mucus and regionally extensive inflammatory lesions in comparison to a naïve WT mouse (Figure 4). Histopathological examination revealed heterogeneity both between βENaC animals, and across lung lobes within individual animals (Figure 4B). A subset of animals, regardless of infection status, displayed severe pathology (Figures 4C, D arrows) characterized by scattered intracellular and extracellular eosinophilic crystals (Figure 4C), emphysema (Supplementary Figure 2B), and an overabundance of airway mucus, all atypical features for WT mice. Crystals could be seen accumulating sporadically throughout the lungs, a characteristic that has been previously reported in βENaC mice (Figure 4C, arrows) and also previously described in (21–23, 26, 34).

In more severe cases of baseline lung inflammation in uninfected βENaC mice, macrophage and eosinophil accumulation formed eosinophilic granulomas around the crystal structures (Supplementary Figure S2). Epithelial damage and hyperplasia were consistently identified and occasionally crystals could be seen breaking down and entering the airway environment. Many lung lobes displayed high levels of lymphocytic infiltration accompanied by neutrophils and macrophages. This finding suggests an active inflammatory response, even in the absence of any known pathogen exposure. In the cases with the most severe pathology, mucus plugging could be observed in cross sections of upper airways by PAS staining (Figure 4D, Additional in Supplementary Figure S3). These findings are consistent with previous work (22, 34) on βENaC transgenic mice describing both airway mucus accumulation and inflammation, features that have not been recapitulated in other traditional CF mouse models. Due to the baseline level of pathological features in uninfected βENaC mice, evidence of additional histological injury was not detectable in infected mice.

### 3.6 *M. abscessus* localization patterns in βENaC lungs

**FIGURE 5:**
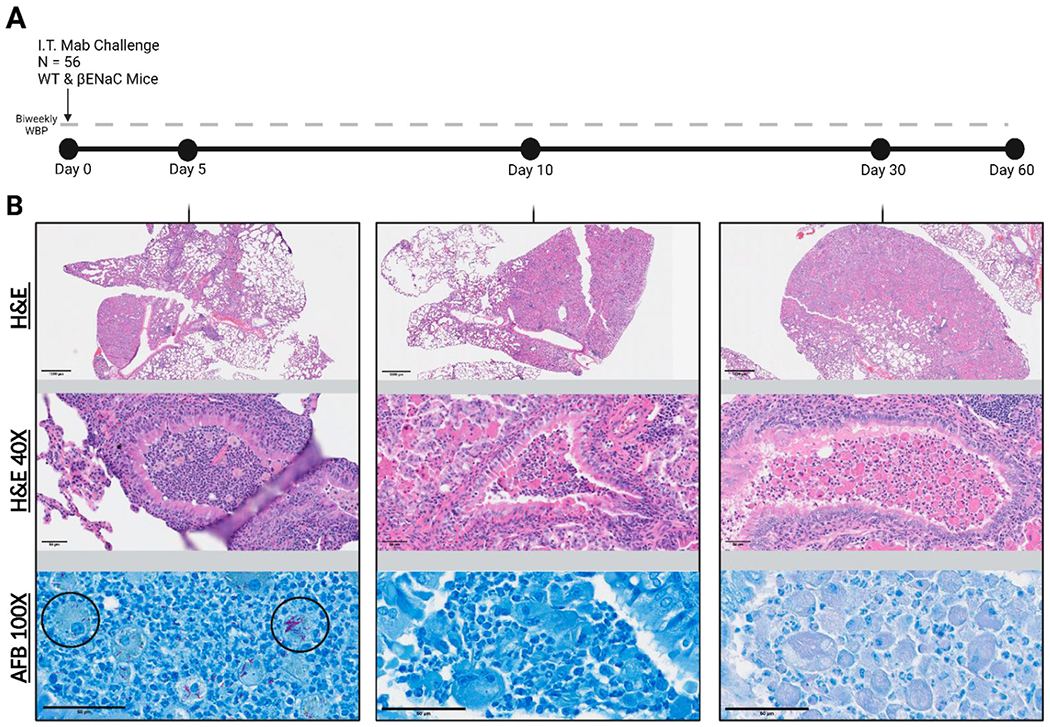
Study timeline and representative βENaC tissue histology. **(A)** 56 mice infected on “Day 0”. Animals were weighed and WBP performed bi-weekly for 60 days post infection. **(B)** Corresponding histological lung sections were periodically collected and stained with H&E or with Ziehl-Neelsen for Acid-Fast Bacilli (AFB). Top images represent a 10x magnified view and middle images are 40x zoomed to showcase βENaC associated lung pathology. The bottom panel shows a 100x AFB stain to investigate *Mab* localization patterns (black circles).

Histopathology was evaluated at endpoints of infection and further examined for the presence and characteristics of acid-fast bacilli (AFB) (Figure 5). Other mouse models of *M. abscessus* pulmonary disease have reported AFB staining patterns in both the acute and chronic phases of infection as free bacilli in the alveoli, and clustered in regions presenting gross lesions (13). Here, examination in WT mice on day 5 post-infection showed extracellular AFB in the upper airways and intracellularly within macrophages. By day 10, AFB was sparsely identified in regions of healthy tissue, located within the lumen of bronchioles. After day 10, bacteria could not be observed through AFB imaging and were only detectable on culture. In contrast to WT mice, *M. abscessus* in the βENaC mice was observed exclusively in the airways. AFB staining on day 5 showed few extracellular bacilli but high numbers of bacteria localized within macrophages, neutrophils, and other phagocytic cells that are more densely populating the bronchioles (Figure 5B). The *M. abscessus* bacilli were trapped in macrophage and neutrophil dense regions, and few bacilli were observed penetrating the bronchial epithelium into the surrounding lung tissue. Closer examination revealed single bacilli within smaller, multinucleated cells and larger clumping of corded *M. abscessus* within the macrophages with a foamy phenotype. By day 10, like the WT mice, the only visible bacteria were sparsely disseminated into the tissues.

## 4 Discussion

The transgenic βENaC mouse, despite its heavy inflammatory and mucosal phenotype, cleared pulmonary bacteria similarly to its C57BL/6N counterpart. Previous studies suggest that CF-like disease in βENaC mice could provide a useful tool for understanding the pathogenesis and clinical presentation of lung disease in immune competent systems (21, 22). Our study demonstrates that the βENaC-Tg C57BL/6N mouse does not facilitate replication of Mab #103, a strain selected for its relevance to CF, rough morphology, and preclinical characterization (12), despite post-clearance WBP data showing a continuous decline in lung function. Notably, three out of the four βENaC animals exhibited early splenic dissemination, a feature that has not been described in other animal models. The specific relationship between sodium absorption and bacterial permeability is not well established in the literature, but it may be possible that the distinctive ionic environment could influence the integrity of the epithelial barrier or bacterial behavior. Alternatively, this observation could be attributed to localized heightened levels of phagocytic cells, particularly neutrophils, with the capacity for systemic circulation. Both hypotheses are likely influenced by many unforeseen factors and further research is warranted to understand these mechanisms. Although these findings diverge from other studies that explored lung infection with *Pseudomonas aeruginosa* and other clinical isolates of *M. abscessus* in βENaC mice, they lay the groundwork for investigating mechanisms involved in NTM pulmonary infection in CF (26, 35).

Since the introduction of human pulmonary function testing in the 1950′s, it has played a pivotal role in assessing various facets of respiratory health (28, 36). However, when it comes to preclinical models, unrestrained whole-body plethysmography in mice remains an underdeveloped tool, particularly for studying markers in mycobacterial lung diseases and therapeutic outcomes. Previous studies suggest that chronic NTM infection is strongly associated with the worsening of lung function over time in CF patients (7), and our plethysmography studies in mice have, for the first time, unveiled a sustained decline in lung function after *M. abscessus* infection that persists after bacterial clearance.

After establishing a suitable protocol for equilibrating the mice in unrestrained WBP chambers, we quantified baseline differences between WT and βENaC mice in the absence of infection, which revealed significant distinctions in their baseline respiratory profiles. WT animals maintained consistently low levels of bronchoconstriction and exhibited a steady upward trend in breath frequency and total volume over the 60-day infection study period, reflecting normal age-related lung maturation (37). Conversely, uninfected βENaC mice displayed consistently elevated levels of bronchoconstriction throughout the study, which did not further increase with *M. abscessus* infection. These baseline differences indicate that the βENaC mice already exhibited a maximum threshold of tolerable bronchoconstriction in their resting state, underscoring the inherent lung dysfunction in this mouse model. Furthermore, both strains of mice had similar increases in MVb, but where the WT animals exhibit a decrease in frequency and increase in volume per breath to achieve this, the βENaC mice only increase breath frequency without increasing the volume per breath.

Following *M. abscessus* pulmonary infection, distinct respiratory changes were observed in both WT and βENaC mice. WT mice cleared the bacteria as reported in previous work (38), but interestingly, these animals demonstrated a constant increase in indicators of bronchoconstriction and a decrease in measures of lung volume. The magnitude of change between the infected and uninfected groups was more pronounced in the WT mice than it was in the βENaC mice. In WT animals, the infection resulted in characteristic alterations associated with progressive lung disease in humans, including increased airway restriction and a decrease in total inhaled volume (39). Notably, breath frequency remained relatively unchanged from uninfected WT mice, but there was a decrease in total inhaled volume accompanied by a significant increase in bronchoconstriction that is easily distinguished in the pre- and post-infection respiratory waveform. In contrast, infected βENaC mice displayed a decline in total lung function that is evidenced by decreases in minute volume. Interestingly, the infection did not impact overall frequency or tidal volume but did significantly increase the time of inhalation and exhalation variables, the major contributing factors to MVb. Pronounced differences were observed in the respiratory waveforms of infected mice of both strains after 60 days, marked by slower and shallower breathes, increased wavelength, and decreased total volume amplitude. These shifts are consistent with physical deconditioning during chronic pulmonary infection and were particularly marked in the βENaC mice. A longer study may reveal whether this trend continues into decompensated respiratory function and premature death.

In addition to quantitative respiratory data, we conducted a histological examination. Regardless of infection status, the postmortem gross pathology of uninfected βENaC mice revealed puffy and discolored lungs with evidence of inflammation. The subsequent histopathological analysis revealed significant heterogeneity among animals and within the same animal between lung lobes. βENaC pathology could be differentiated by an excess of airway mucus globules and scattered eosinophilic crystals—features atypical for WT mice. In more severe cases, eosinophilic granulomas formed around the crystal structures, accompanied by epithelial damage, hyperplasia, and occasional breakdown of the crystals into the airway environment. Aligned with previous reports on βENaC transgenic mice, many lobes exhibited leukocyte infiltration rich in neutrophils and macrophages, indicative of an ongoing inflammatory response irrespective of infection status (21, 25).

Several factors may account for the ability of βENaC mice, despite their CF-like presentation, to clear *M. abscessus* pulmonary infection. Persistent airway neutrophilia, a well-established characteristic in βENaC mice, is evident in this study. Neutrophils have both intracellular and extracellular mechanisms to kill *M. abscessus* (40). Our AFB imaging reveals that the majority of *M. abscessus* is harbored within phagocytic airway neutrophils and macrophages, in contrast to the phagocytic dysfunction typically associated with CFTR mutations (41). This finding further implies a potential role for functional CFTR in supporting neutrophil-mediated *M. abscessus* killing in βENaC mice. The persistence of neutrophilia was consistently observed in animals older than 8 weeks and was sustained in mucus-plugged airways throughout the study, regardless of infection status. The exact implications of this neutrophil response in *M. abscessus* remains complex and unclear. While neutrophils are evidently involved in early microbial clearance here, their antibacterial functions, which include phagocytosis, degranulation, and neutrophil extracellular trap (NETs) formation, are also strongly associated with irreversible airway damage (42). Further work is warranted to explore the potential connection between *M. abscessus* pulmonary challenge and neutrophil-mediated respiratory decline in mice. The βENaC mouse model offers valuable insights into respiratory pathology and inflammatory responses but is not a suitable model for studying chronic Mab pulmonary infection and does not truly recapitulate the mechanisms of mucus secretion in human lungs. Furthermore, while the pulmonary clearance of *M. abscessus* 103 rough strain was observed in this study, it is important to note that this outcome may not be consistent across all subspecies and morphologies of *M. abscessus*. If the study were repeated using *M. abscessus* ATCC 19977, which exists in both smooth and rough morphologies, we hypothesize a slower rate of clearance from the smooth strain due to the known differences in intracellular processing between these two forms (43), but given the functionality of airway phagocytes in the βENaC mice, it is reasonable to expect that bacterial elimination would still occur. Additionally, the βENaC mice may have a propensity to sustain chronic infection with the slow growing mycobacteria in the *M. avium* complex (MAC). However, the already histologically dense tissue in thes mice can inhibit or mask the formation of MAC-related lesions, and the C57BL/6 background is not as suitable for modeling pulmonary MAC when compared to the BALB/c mouse (44).

WBP is a non-invasive method for respiratory function measurements that lends itself to longitudinal studies with large sample sizes. Importantly, WBP measurements encompass the entire respiratory system, including the nasal passage, which may contribute to data variability but ultimately does not require intubation. Other methods, notably the FlexiVent [SCIREQ©] system, have been extensively characterized in mice, but are challenging to implement for longitudinal *in vivo* studies and require more invasive techniques (45, 46). We chose to employ WBP in this context due to its capacity for larger scale studies and its potential for highlighting long-term trends in total respiratory function. With this data, we aim to identify additional markers of respiratory decline, in conjunction with animal scoring and weight acquisition, for monitoring the progression of pulmonary mycobacterium infections.

Longitudinal WBP excels at detecting subtle changes in respiratory patterns, offering critical insights into the role of NTM in the onset and progression of bronchiectasis. Parameters such as breath frequency, minute ventilation, and airway resistance (reflected as Penh) can be closely monitored to track respiratory dysfunction, mirroring the measures taken to monitor lung function in human bronchiectasis. This approach holds promise for identifying early markers of disease progression and assessing the effectiveness of therapeutic interventions, further clarifying the role of NTMs in the development and exacerbation of bronchiectasis. Future studies should focus on the long-term respiratory decline associated with NTM infections, including the effects of repeated Mab exposure on lung function.

The changes in both βENaC mice and WT mice respiratory function following *M. abscessus* infection provide a valuable platform for studying markers of pulmonary infection and assessing candidate therapies. These quantifiable measures of respiratory function could serve as benchmarks for evaluating the efficacy of potential treatments in the context of mycobacterial lung diseases and other related pulmonary disorders. These results indicate that lung function is impaired during and after infection with *M. abscessus* in both CF and non-CF like environments, and that the long-term effects of NTM exposure should be further explored in the context of respiratory health. Future studies will explore the utility of WBP in assessing therapy efficacy for mycobacterial pulmonary infections.

## Supplementary Material

Supplementary Figures

## Data Availability

The raw data supporting the conclusions of this article will be made available by the authors, without undue reservation.

## Abbreviations:

*M. abscessus*: *Mycobacterium abscessus*
CF: Cystic Fibrosis
ENaC: Epithelium Sodium Channel
βENaC: β-Subunit ENaC Overexpressing
WBP: Whole Body Plethysmography
NTM: Non-Tuberculous Mycobacterium
CFTR: Cystic Fibrosis Transmembrane Regulator
NET: Neutrophil Extracellular Trap
WT: Wild-type
H&E: Hematoxylin and Eosin
AFB: Acid Fast Bacilli
PAS: Periodic Acid-Schiff
CFU: Colony Forming Units
OADC: Oleic Acid; Albumin; Dextrose; Catalase
AUC: Area Under Curve
Ti/Te: Time of Inspiration/Expiration
TVb: Tidal Volume per Breath
MVb: Minute Volume per Breath
f: Frequency
EIP/EEP: End Inspiratory/Expiratory Pause
Penh: Index of Bronchoconstriction
BPM: Breaths per Minute
