# Supplementary material for: Mycobacterium abscessus pulmonary infection and associated respiratory function in cystic fibrosis-like βENaC mice
Source: Front Tuberc. 2024 Oct 30;2:1473341. doi: 10.3389/ftubr.2024.1473341 (PMC11822858; doi:10.3389/ftubr.2024.1473341)
Supplement: Supplementary file 1 [file Data_Sheet_1.PDF]

Supplementary

Figure S1: Characteristics of Mab pulmonary infection in WT mice

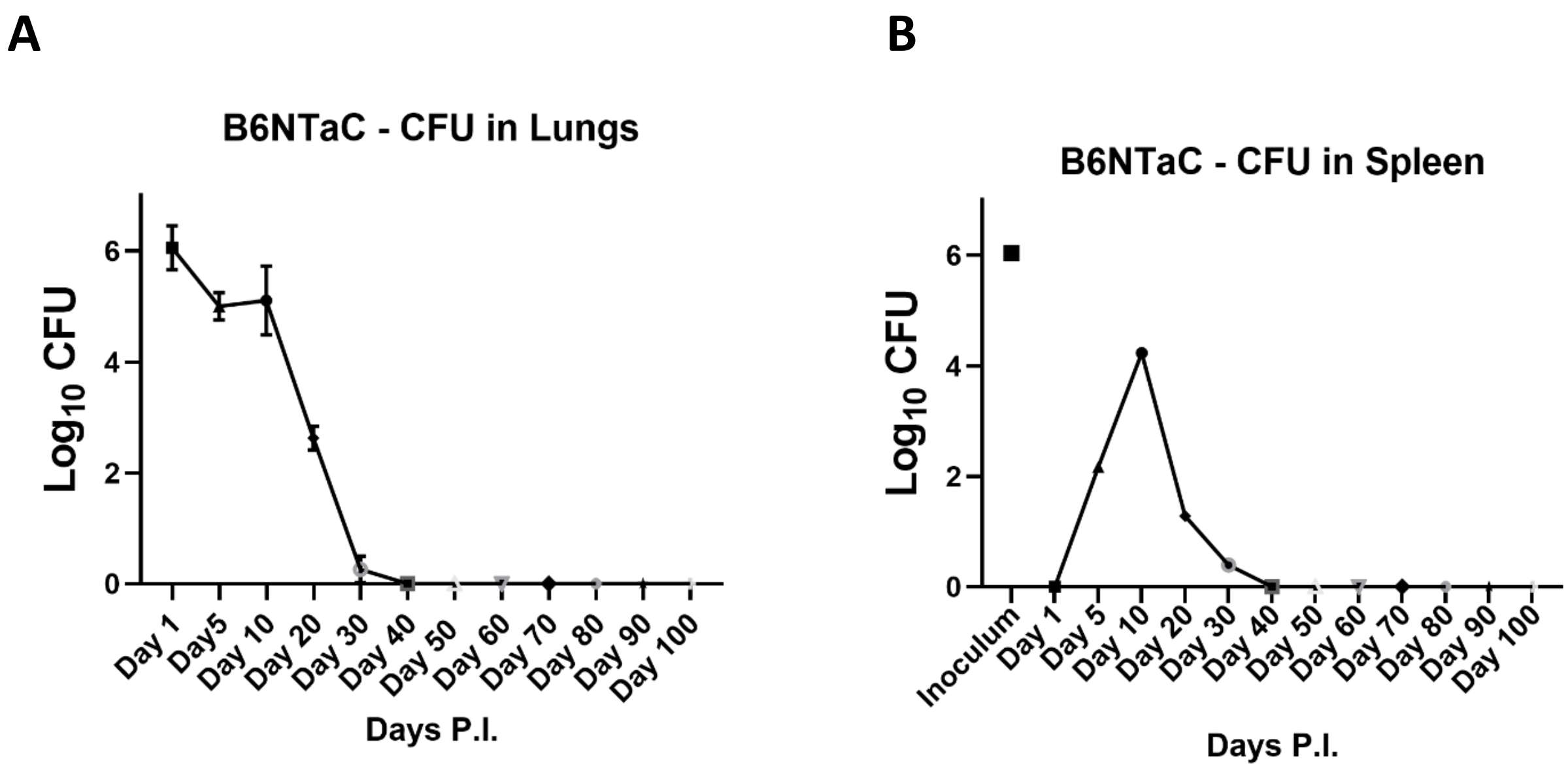

Figure S1. Prior to the studies, n = 75 wildtype C57BL/6N (B6NTac) mice were infected with *M. abscessus* #103 to characterize pulmonary infection in the background animals. (A) Shows whole and left lung, and whole spleen (B), adjusted Log_{10}CFU on the y-axis and days elapsed (or inoculum CFU) following infection on the x-axis. All animals in the study cleared the bacteria to below the limits of detection by day 40 for both the lung and spleen.

Figure S2: βENaC Related Histology

A

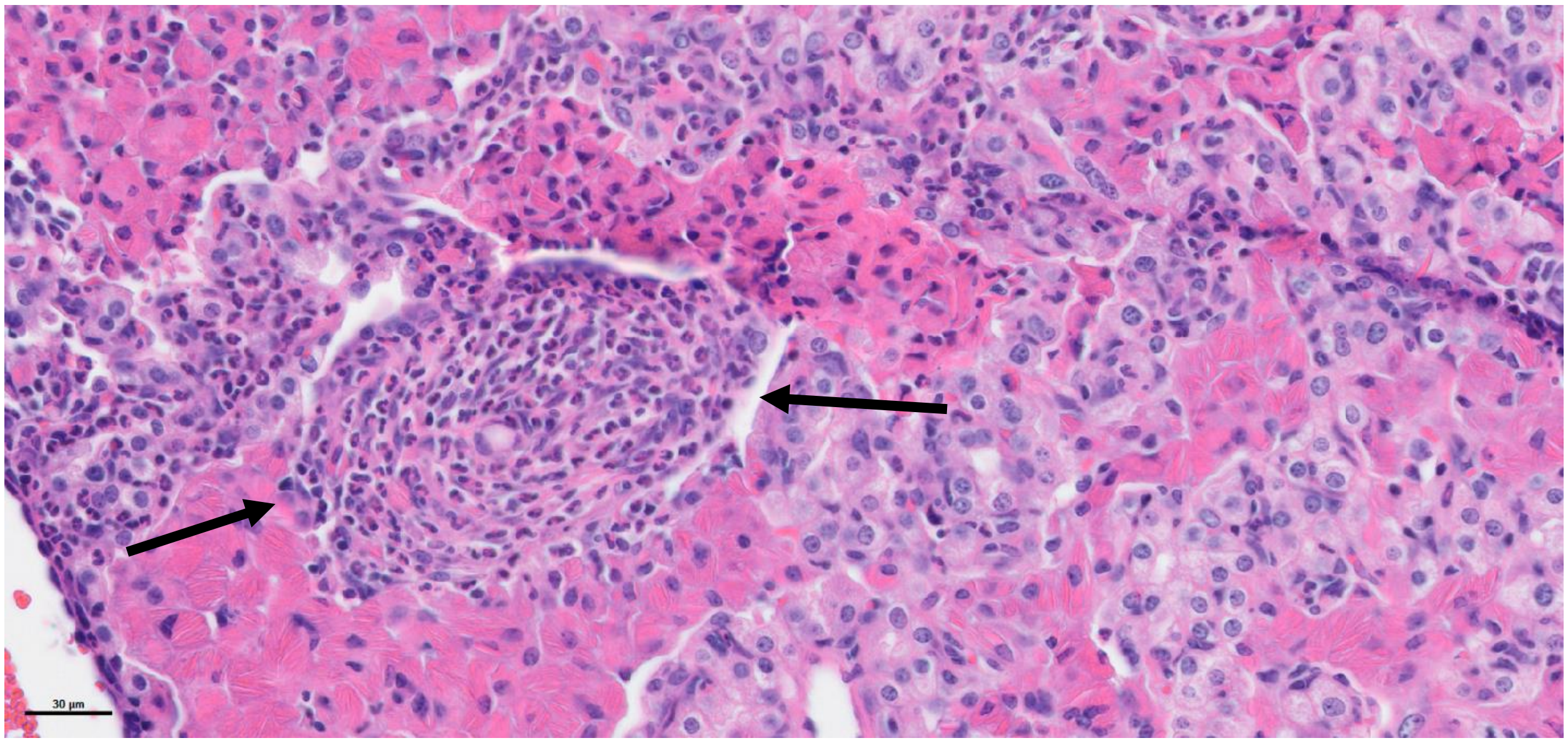

B

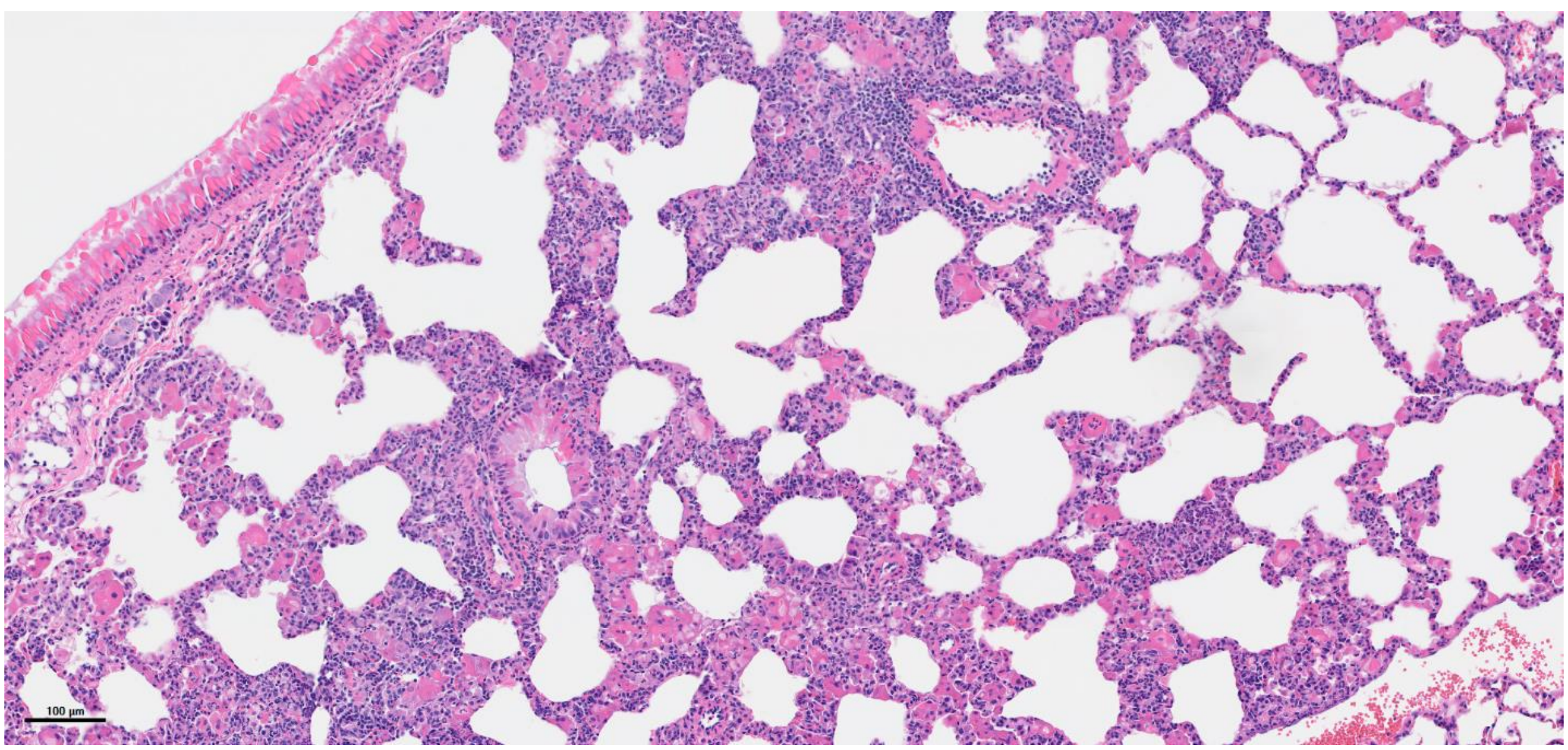

Figure S2: H&E images that are representative of typical features described in section 3.5 and 3.6. (A) is showing a severe case of lymphocytic and eosinophilic accumulation around crystal structures. Areas of bright pink are clusters of eosinophil crystals, and the black arrows are indicating the accumulation of purple stained lymphocytes. (B) represents an area with damaged alveoli. The inner walls of each individual alveolar space are small or ruptured, creating larger air spaces. Amongst this tissue, there is also increased macrophage and neutrophil infiltration.

Figure S3: PAS Staining of mucus abundance

A

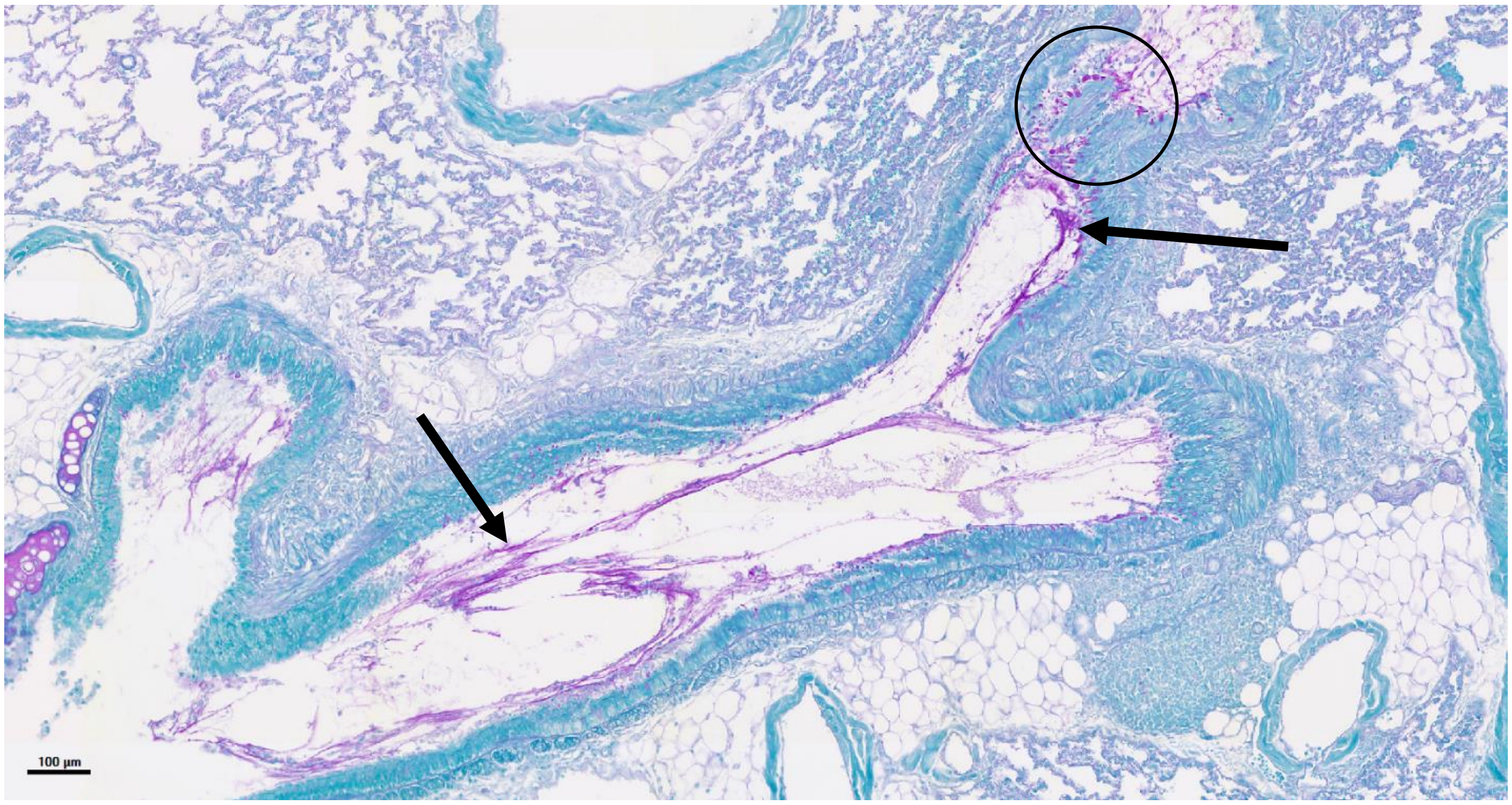

B

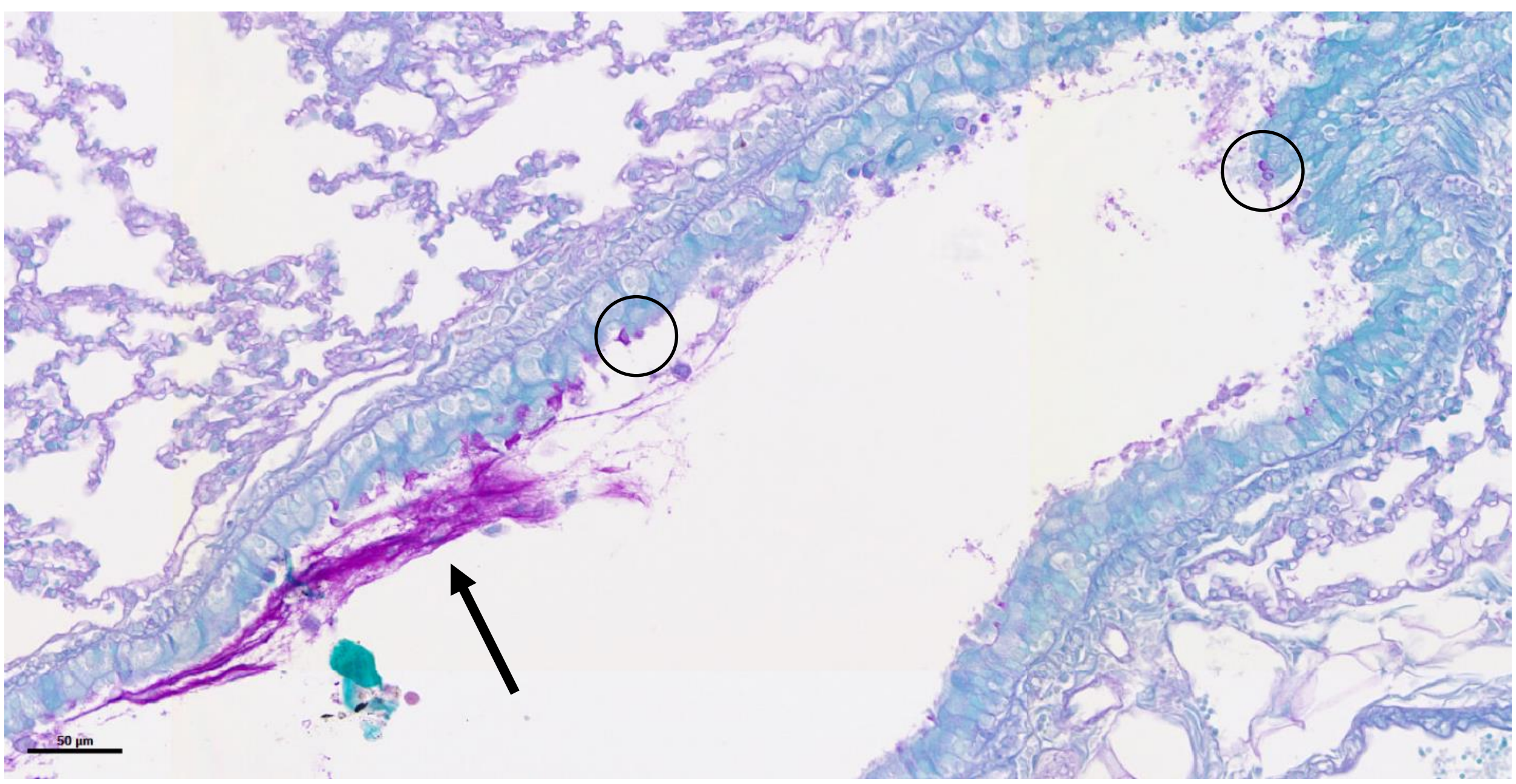

Figure S3: PAS staining of mucus abundance as indicated in main text sections 3.5 & 3.6. (A) shows an airway with mucus plugging in purple. The arrows indicate regions of PAS staining which are atypical in a WT mouse. (B) shows an airway with regional mucus buildup (arrows). In both images, black circles highlight the abundance of mucus globules which are absent in WT animals.
